# Informed consent and minors

**DOI:** 10.1186/1824-7288-41-S2-A51

**Published:** 2015-09-30

**Authors:** Andrea Nicolussi

**Affiliations:** 1 Università Cattolica del Sacro Cuore, Istituto Giuridico, Milan, Italy

## 

The provision of informed consent by a patient should be the end point of a process of engagement in which one or more health practitioners have supported the patient to come to an informed decision to agree to the healthcare offered. Informed consent is a means to comply with the principle of respect for the person in a healthcare context. This act can be alternatively interpreted according to a libertarian perspective or according to a more solidaristic one [1]. According to the former perspective, informed consent is tightly linked to the idea that every person is the proprietor of their own body with which they have the absolute right to decide whatever they want done to it. This view leads to the construction of the patient physician relationship founded on a contractual basis. According to the latter perspective, respect for the person implies a special care and not, indifference, so that consent implies the due engagement of the person in the decision process as the very etymology of the word consent suggests. Respect for a person certainly means respect for his or her autonomy, but the former concept does not correspond necessarily with the latter, especially if autonomy is not interpreted within a framework of solidarity and the patient is not sufficiently mature. Otherwise respect would slide into indifference.

As for minors, the Oviedo convention prefers to use the word *authorisation* instead of the common expression *consent on behalf of the child*. In fact, authorisation relates to the concept of a third authority, such as parental responsibility, which implies a different framework than that of informed consent. This is the concept of the child's best interests as the fundamental criterion of making decisions regarding children. Since informed consent can be seen as an expression of personal choice, it can only be given by the person who is to be provided with health care. Moreover, the Oviedo convention requires that the opinion of the minor shall be taken into consideration. As a result, the decision-making process involves three categories of subjects: the physician who proposes the therapy, the parents who give authorisation and the minor whose opinion “is an increasingly determining factor in proportion of his or her degree of maturity” [2-5]. Further problems can derive from the fragile condition of modern-day families and the related difficulty in parents agreeing on any given issue.
